# Detecting subject-specific activations using fuzzy clustering

**DOI:** 10.1016/j.neuroimage.2007.03.021

**Published:** 2007-07-01

**Authors:** Mohamed L. Seghier, Karl J. Friston, Cathy J. Price

**Affiliations:** Wellcome Trust Centre for Neuroimaging, Institute of Neurology, 12 Queen Square, London WC1N 3BG, UK

**Keywords:** RFX, random-effect analysis, FCM, fuzzy c-mean clustering, FCP, fuzzy clustering with fixed prototypes, D, similarity metric, U, degree of membership, G, degree of contribution, Functional magnetic resonance imaging, Overt object naming, Inter-individual variability, Outliers, Atypical activations, Fuzzy clustering, Second-level analysis

## Abstract

Inter-subject variability in evoked brain responses is attracting attention because it may reflect important variability in structure–function relationships over subjects. This variability could be a signature of degenerate (many-to-one) structure–function mappings in normal subjects or reflect changes that are disclosed by brain damage. In this paper, we describe a non-iterative fuzzy clustering algorithm (FCP: fuzzy clustering with fixed prototypes) for characterizing inter-subject variability in between-subject or second-level analyses of fMRI data. The approach identifies the contribution of each subject to response profiles in voxels surviving a classical *F*-statistic criterion. The output identifies subjects who drive activation in specific cortical regions (local effects) or in voxels distributed across neural systems (global effects). The sensitivity of the approach was assessed in 38 normal subjects performing an overt naming task. FCP revealed that several subjects had either abnormally high or abnormally low responses. FCP may be particularly useful for characterizing outlier responses in rare patients or heterogeneous populations. In these cases, atypical activations may not be detected by standard tests, under parametric assumptions. The advantage of using FCP is that it searches all voxels systematically and can identify atypical activation patterns in a quantitative and unsupervised manner.

## Introduction

In functional neuroimaging, group analyses are used to assess effects of interest at the population level (e.g., Benali et al., 2003; Bosch, 2000; Friston et al., 1999; Lazar et al., 2002; McNamee and Lazar, 2004; Penny et al., 2003; Svensen et al., 2002). They assess the reliability or consistency of responses across individuals, in relation to the inter-subject variations that are assumed to be random. In this paper, we are concerned with the identification of ‘outliers’ in group analyses, i.e., data points that deviate markedly (either very high or very low activation) in one subject, compared to the others (Grubbs, 1969; Rasmussen, 1988). Outliers^1^ inflate the variance and move the mean towards the outlier which is problematic for both parametric and nonparametric statistics (e.g., Gastwirth, 1966; Zimmerman, 1994). Here, we present a new approach for identifying the contribution of each subject to group activations in functional imaging studies.

**Fig. 1:**
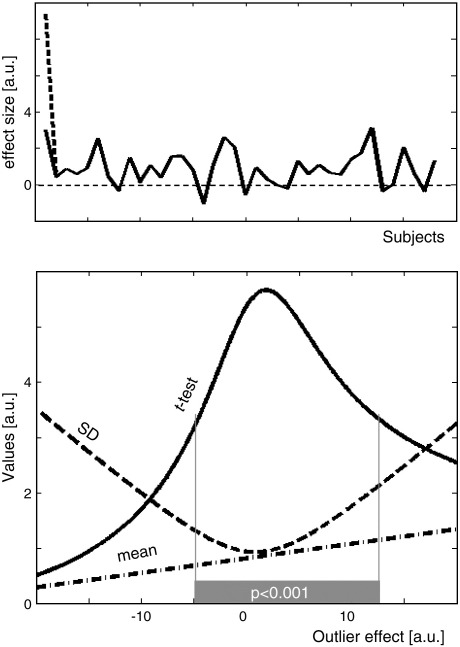
(Top) Activation level in all subjects at a given voxel. The activation level in subject 1 is artificially modified to perturb the mean and the standard deviation (SD) across subjects (e.g., dashed line). (Bottom) The influence of activation level in subject 1 (*x*-axis) on the mean (dash-dot line), SD (dashed line) and *t* values (solid line) is illustrated. Significant effect across subjects (*t* values at *p* < 0.001) is indicated by a horizontal gray bar.

The effect of an outlier on the group activation at a given voxel is illustrated in Fig. 1 using simulated data. When the effect size of one subject (subject 1 in Fig. 1) is increased or decreased, it perturbs the group mean and dispersion across subjects. In this example, the heightened response from one subject increases the variance and reduces the significance of the group effect (i.e., lowers *t* values). True group effects (i.e., activations that are consistent across subjects) can therefore be lost if one subject has an atypical response.

In real data, outliers can reflect technical artefacts and less obvious problems such as sampling from mixtures of populations (for reviews see Beckman and Cook, 1983; Osborne and Overbay, 2004). The importance of identifying the influence of outliers on group effects has been addressed extensively (e.g., Beckman and Cook, 1983; Osborne and Overbay, 2004) and several techniques have been proposed to identify, modify, or remove outliers, before performing statistical tests (Barnett and Lewis, 1978; Beckman and Cook, 1983; Belsley et al., 1980; Bradlow and Zaslavsky, 1997; Chaloner and Brant, 1988; Davies and Gather, 1993; Gastwirth, 1966; Grubbs, 1969; Hawkins, 1980; Hocking, 1976; Huber, 1981; Rousseeuw and Leroy, 2003; Shapiro and Brady, 1995). Outlier removal furnishes inferences that are more robust to parametric assumptions. However, in fMRI, only a few attempts have been made to assess the contribution of outliers to the mean effect (Kherif et al., 2003) even though it is known that inter-subject variability in activations is higher than within-subject or between-session variability (Smith et al., 2005; Wei et al., 2004).

In this context, we present a new approach based on the classification of all voxels according to the clustering principle of fuzzy logic (Zadeh, 1965). Fuzzy logic classification (Bezdek, 1981) has been employed previously in fMRI data analysis (e.g., Baumgartner et al., 1998; Buerki et al., 2003; Fadili et al., 2000; Golay et al., 1998; Jahanian et al., 2004; Windischberger et al., 2003), but only for data-driven first-level analyses (e.g., time series classification of all voxels without *a priori* knowledge). Our approach, fuzzy clustering with fixed prototypes (FCP), is specifically tailored to the exploration of inter-subject variability at the second level, where each data point represents an effect (i.e., summary statistic or contrast) from a single subject. By conducting the analysis at the second level, our method also differs from others by being non-iterative and hypothesis-driven with predefined clusters (i.e., prototypes). We demonstrate that FCP can identify regional group effects that are driven or hidden by high or low activation in one subject.

## Methods

### Subjects

Thirty-eight healthy right-handed subjects (13 males, 25 females, 32 ± 20 years) gave written informed consent to participate in this study. Subjects were native English speakers, had normal or corrected-to-normal vision, with no history of neurological or psychiatric disorders. This study was approved by the Medical Ethics Committee of the Institute of Neurology.

### Paradigm and stimuli

There were two conditions of interest: (a) an activation task that involved object naming and (b) a baseline task that controlled for visual and articulatory processing by requiring participants to articulate “1, 2, 3” in response to pictures of meaningless non-objects. To facilitate task switching, the conditions were blocked with twelve pictures (objects or non-objects) per block. Within a block, the twelve pictures were presented as four sequential stimuli (one stimulus per 4.3 s) with three pictures per stimulus, one above and two below. The participants were asked to name the objects in the same order (top, bottom left, bottom right) or to say “1, 2, 3” to the non-objects while looking at the top, bottom left and bottom right picture. Over the experiment, there were 32 object naming stimuli (96 pictures) and 16 non-object stimuli (48 pictures). These conditions of interest were interspersed with fixation, reading aloud and saying “1,2,3” to meaningless symbols. The total scanning time for all conditions was 12 min in two separate six-minute sessions.

To ensure that the task was understood correctly, all subjects were provided with detailed instructions and underwent a short training session before entering the scanner. To minimize artefacts from head motion, subjects were instructed to whisper their response with minimal mouth movement. Subject responses were recorded with an in-house MRI-compatible auditory recording system.

### MRI acquisition

Data were acquired on a 1.5 T Siemens system (Siemens Medical Systems, Erlangen, Germany). Functional imaging comprised an EPI GRE sequence (TR/TE/flip = 3600 ms/50 ms/90°, FOV = 192 mm, matrix = 64 × 64, 40 axial slices with 3 × 3 × 3 mm^3^ voxel size). The multi-slice volume was positioned on sagittal scout images. Functional scanning was always preceded by 14.4 s of dummy scans to insure tissue steady-state magnetization.

### Data analysis

Data processing and statistical analyses were performed with the Statistical Parametric Mapping SPM2 software package (Wellcome Department of Imaging Neuroscience, London UK, http://www.fil.ion.ucl.ac.uk/spm/). All functional volumes were spatially realigned, un-warped, normalized to the MNI space, and smoothed with an isotropic 6-mm FWHM Gaussian kernel, with resulting voxels size of 2 × 2 × 2 mm^3^. Time-series from each voxel were high-pass filtered (1/128 Hz cut-off) to remove low-frequency noise and signal drift. The pre-processed functional volumes of each subject were then submitted to a fixed-effects analysis, using the general linear model at each voxel. Each stimulus onset was modeled as an event encoded in condition-specific ‘stick-functions’. The resulting stimulus functions were convolved with a canonical hemodynamic response function to form regressors for the linear model. Our contrast of interest was the main effect of object naming relative to the non-object baseline. The appropriate summary or contrast image (i.e., a contrast of maximum likelihood parameter estimates) was then entered into a second-level analysis (i.e., random-effects analysis) to enable inferences about the population from which our subjects were drawn. From this second level analysis, we generated a statistical parametric map of the *F* statistic at each voxel SPM{*F*}, which characterized differences in activation (activations and deactivations) for object naming relative to the non-object baseline. The SPM of the *F*-statistic was used to identify candidate voxels for subsequent fuzzy clustering. It would be perfectly possible to include all brain voxels in the clustering analysis (i.e., without selection based on the *F*-statistic); however, one is usually interested in detecting outlier responses in regions that are typically engaged by the experimental paradigm. The clustering itself used the contrasts summarizing the activation for each subject at the candidate voxels. In the absence of outliers, we would expect these contrast values to be normally distributed, by central limit theorem.

### Non-iterative fuzzy clustering with fixed prototypes (FCP)

**Fig. 2:**
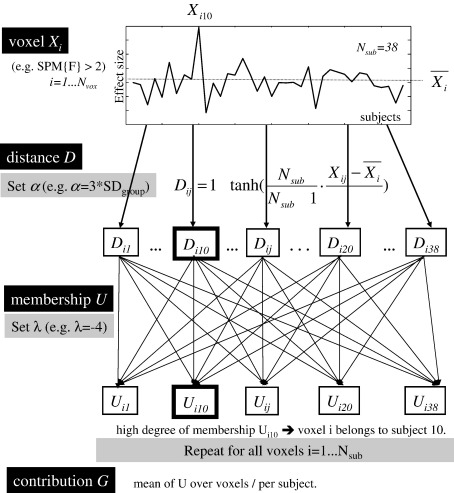
Schematic illustration of the FCP algorithm.

The algorithm for FCP is adapted from Bezdek's fuzzy c-mean (FCM) clustering approach (Bezdek, 1981; Bezdek et al., 1997). In contrast to FCM, FCP is a non-iterative method that uses prototypes (i.e., clusters) that are fixed *a priori*, this means that the number of clusters does not have to be estimated and the procedure can be implemented non-iteratively. See Fig. 2 for a schematic illustration of the FCP algorithm.

In practice, we select *N*_vox_ voxels that we want to assign to *C* clusters. Here, we included all voxels with *F* > 2.0 in the second-level analysis (about 85,000 voxels). As outlier subjects are unknown, all subjects represent plausible classes in our algorithm. Therefore, the number of clusters (i.e., prototypes) is equal to the number of subjects *C* = *N*_sub_. This means that each cluster represents the contribution of the corresponding subject to the mean effect at the voxel level. Each voxel *i* has a vector *X*_*i*_ of *N*_sub_ values that correspond to the contrast (i.e., activation) for each subject. The resemblance between each voxel *i* and each cluster (prototype) *j* is characterized by a “similarity metric” *D*_*ij*_. The degree of membership *U*_*ij*_ is calculated for each voxel *i* by comparing *D*_*ij*_ for each cluster *j* to all other clusters.

### Similarity metric *D*

We quantify the similarity metric *D*_*ij*_ between a voxel *i* and prototype *j* as:

(1) $$D_{ij}=1-\tanh(\frac{N_{\text{sub}}}{N_{\text{sub}}-1}\cdot \frac{X_{ij}-{\overset{¯}{X}}_{i}}{\alpha })=1-\tanh(N_{\text{sub}}\cdot \frac{{\overset{¯}{X}}_{i}-\overset{¯}{X_{i}^{\neq j}}}{\alpha })$$

The real constant *α* is a “tuning” parameter that can be adjusted to control the sensitivity of the method to outlier values (see below). tanh is the hyperbolic tangent, *X*_*ij*_ is the effect for the *j*-th subject at voxel *i*, *X¯*_*i*_ is the mean over subjects and *X*_*i*_^*≠j¯*^ is the mean effect without subject *j*. Accordingly, the similarity metric can be interpreted as (i) a measure of how far subject *j* is from the group mean, scaled by *α* or; (ii) a measure of how the mean effect of the group is perturbed when subject *j* is excluded. The latter perspective is important and suggests that our algorithm is formally similar to regression diagnostic methods that assess the extent to which a particular data point influences the model, by determining the change when that point is omitted; for example, the Cook's *D*-distance (Cook and Weisberg, 1982) and the DFFITS/DFBETAS statistics (Belsley et al., 1980). Note that other resemblance metrics have been employed in previous studies with standard fuzzy classification, including the hyperbolic correlation measure (for more details, see Fadili et al., 2000; Golay et al., 1998).

**Fig. 3:**
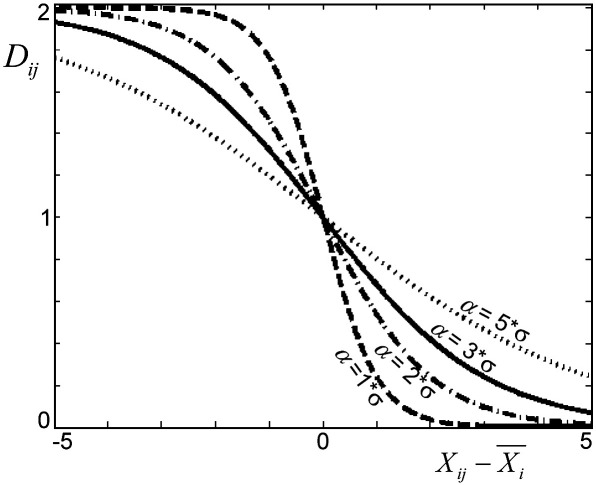
The influence of the parameter *α* on the smoothness of the distance *D*. Values of *α* of 1, 2, 3 and 5 are illustrated in this graph. Solid line represents the value of *α* used for the rest of the paper.

To illustrate the effect of *α*, we considered a voxel *i* with a given mean effect *X¯*_*i*_ and a standard deviation equal to one. Fig. 3 illustrates the influence of parameter *α* on *D*. Increasing *α* leads to “smooth” *D* values, suggesting that *α* can be considered as a “smoothness” parameter. Critically, in order to keep the method independent of the scaling of *X*, we set *α* equal to 3 · *α*, where *α* is the standard deviation of the group (i.e., standard deviation over all voxels and all subjects). Voxels that are driven by high positive activation (i.e., large effects) are identified with a positive *α* value (3 · *α*) and voxels that are driven by low or negative effects (e.g., deactivation) are identified with a negative *α* value (− 3 · *α*).

### Degree of membership *U*

The similarity metric *D* is then used to quantify the degree of membership *U*_*ij*_ of voxel *i* to cluster *j* according to the following equation:

(2) $$U_{ij}=\frac{D_{ij}^{\lambda }}{\sum_{j}D_{ij}^{\lambda }}$$

**Fig. 4:**
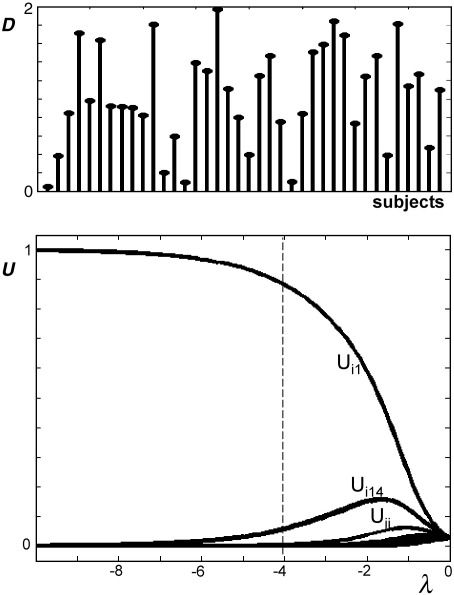
(Top) Typical *D* values when for instance subject 1 is far from the group. Subject 1 has therefore a lower *D* value than other subjects. (Bottom) the influence of parameter *λ* on *U* values. For instance, for a given voxel *i*, subject 1 has high activation level compared to other subjects, which means that this voxel *i* has high degree of membership *U*_*i*1_ to subject 1. When *λ* tends to − ∞ the classification becomes hard (*U*_*i*1_ = 1, and *U*_*ij*_ = 0 for *j* ≠ 1). When *λ* goes to 0 the classification is fuzzy (all *U*_*ij*_ are near to 1/*N*_sub_). For intermediate *λ* values, *U*_*ij*_ is a continuum between 0 and 1. In our case, *λ* is fixed at − 4 (dashed line).

The parameter *λ* is a negative number that represents the degree of fuzziness (e.g., Fadili et al., 2000) or the defuzzification parameter (e.g., Dimitriadou et al., 2004) as defined in the FCM approach. The influence of fuzziness on the clustering has been explored in previous studies (e.g., Bezdek, 1981; Fadili et al., 2000, 2001; Krishnapuram and Keller, 1993): when *λ* tends to − ∞ the classification becomes hard and *U*_*ij*_ takes the value 0 (voxel *i* is not a member of cluster *j*) or 1 (voxel *i* belongs to cluster *j*) but when *λ* goes to 0 the classification is fuzzy (*U*_*ij*_ is near to 1/*N*_sub_). Fig. 4 illustrates the influence of *λ* on *U* with a range of *D* values. In our approach, classification was fuzzy (*U*_*ij*_ was a continuous number between 0 and 1) when *λ* was between − 8 and − 2 (Fig. 4). We held *λ* constant at − 4 (see below for more details).

To localize regions that are driven by subject *j*, the *j*th column of *U* is thresholded at 0.3 and displayed on a normalized anatomical volume. These maps identify the regions that are driven by a particular subject.

### Contribution coefficient *G*

We assess the contribution of each subject to the distributed response by computing the following coefficient:

(3) $$G_{i}=\frac{1}{N_{\text{vox}}}\sum_{i}U_{ij}$$

This coefficient *G*_*j*_, computed for each cluster, is the relative proportion of the brain volume that belongs to the *j*-th subject (note that *G*_*j*_ sums to one). In hard classification (i.e., *λ* → − ∞), *G*_*j*_ is simply the proportion of voxels that belong to the *j*-th subject. In fuzzy classification, it is an estimation of how each subject dominates the observed data in a ‘global’ and “relative” way. In this sense, *G*_*j*_ reflects the ‘global’ contribution of the *j*-th subject relative to other subjects. The profile of *G*_*j*_ over subjects allows one to detect subjects who dominate in their contribution to the overall activations observed.

### Simulations with FCP method

Before applying FCP to real fMRI data, we performed several simulations to assess (i) the sensitivity, (ii) the specificity, (iii) the influence of the parameter *λ* and (iv) the distribution of *G* values in a group with or without outlier subjects.

To assess the specificity and the sensitivity of FCP, we generated receiver operating characteristic (ROC) curves (Metz, 1978; Sorenson and Wang, 1996) at different values of the parameter *λ*. ROC curves encode the dependence of the true positive rate (sensitivity) on the false positive rate (one minus specificity) for different thresholds on the degree of membership *U*. Practically, 100,000 artificial voxels and 38 subjects were generated from a unit normal distribution (mean = 0, *σ* = 1). In a particular subject (e.g., subject 20), a proportion *q* of voxels (i.e., *q* = 5%, five thousands voxels) was sampled from a different normal distribution (mean = 3, *σ* = 1) and considered as a true positive (i.e., a true outlier). We then ran FCP on these simulated data with *σ* = 3*. G* values were assessed for each subject. This procedure was repeated for several values of *λ* = − 1, − 2, − 3, − 4, − 6, − 8, − 10, − 20 and − 40 and for different outlier distributions (mean = 3, 4 and 5).

To assess sensitivity we treated one subject (e.g., subject 20) as an outlier. Two different proportions were used: *q* = 1% (1000 outlier voxels) and *q* = 0.2% (200 outlier voxels). For both proportions, 10,000 simulations were performed. We assessed specificity by generating the null distribution of *G* with *λ* = − 4; we generated 100,000 voxels and 38 subjects from a normal distribution (mean = 0, *σ* = 1) (i.e., with no outliers). This procedure was repeated 10,000 times to provide samples of *G* under the null hypothesis of no outliers. We repeated the simulations but with different number of subjects *N*_sub_ (with 10,000 iterations for each group size *N*_sub_). In these analyses, *N*_sub_ varied from 10 to 50.

Although these simulations used normally distributed data, clustering with fuzzy logic does not assume normality of the data. The only assumption we made was the existence of outliers far from the group mean (irrespective of the distribution of the population) and simply translated this assumption to a resemblance measure *D*.

## Results

### Simulated data

#### Influence of *λ*

**Fig. 5:**
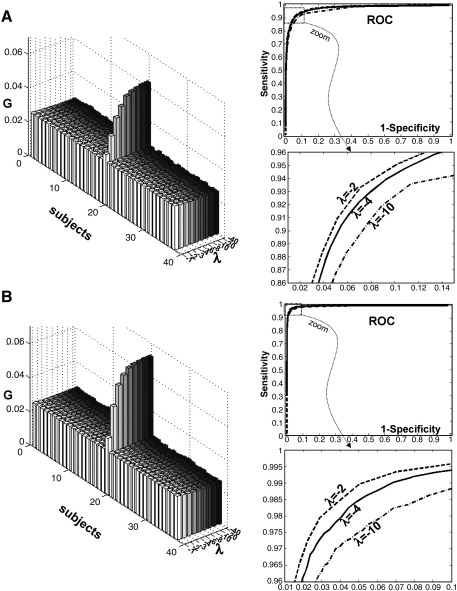
A—(left) *G* values on simulated data with subject number 20 as an outlier (with 5% of voxels set to 3*σ* above the mean of the group). *G* values are computed at different *λ* values. (Right-top) ROC curves on simulated data with different *λ* values. (right-bottom) zoom on ROC curves. B—The same as panel A but with outlier voxels set to 4*σ* from the mean. See the Methods section for more details.

As shown in Fig. 4, parameter *λ* affects the assessment of degree of membership *U*. Therefore, the coefficient *G* also depends on this parameter. Fig. 5A illustrates the influence of *λ* on *G* values when an outlier subject contained voxels with an effect at three standard deviations from the mean. When *λ* is small in absolute value (e.g., − 1 or − 2), the difference between the *G* value of the outlier subject and the *G* values of other subjects is low, suggesting low discrimination. This is due to the fact that *U* values are very similar (near to 1/*N*_sub_) when *λ* tends to zero (Fig. 4). On the other hand, the ROC analyses of sensitivity and specificity at the voxel level showed low sensitivity when *λ* is too high in absolute value (e.g., − 10). This might be explained by the fact that voxel clustering becomes categorical (i.e., *U* = 0|1) when *λ* tends to − ∞ and can miss outlier voxels in the presence of noise, as illustrated by our simulations. Empirically, intermediate values of *λ* (e.g., − 4) appear the best compromise between high sensitivity at the voxel level and high sensitivity at the subject level. The same conclusions were reached when the effect of an outlier is very far from the mean of the group (see Fig. 5B). When applying FCP to real data, we therefore set *λ* equal to − 4.

#### Distribution of *G*

**Fig. 6:**
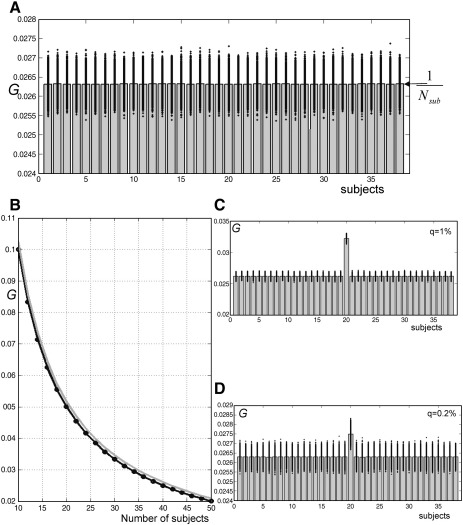
(Top) Distribution of *G* values on simulated data when all subjects are sampled from the same distribution (no outliers). Bars represent mean *G* values over 10,000 realizations for each subject, and each dot represents the *G* value for each realization (10,000 dots per subject). *G* values under null hypothesis are near to the theoretical value of 1/*N*_sub_. (Bottom-left) mean of *G* values (solid line) + 5*σ* (gray line) over all simulations for different sample sizes (*N*_sub_ from 10 to 50 subjects). (Bottom-right) The same computations as in top but with subject number 20 having a proportion of *q* = 1% or *q* = 0.2% of voxels with outlier values.

Fig. 6A showed the distribution of *G* when all subjects are comparable (i.e., no outliers). Over 10,000 simulations, *G* values are stable with a mean equal to 1/*N*_sub_ (0.026) and a standard deviation of 0.0002. Consequently, we can define a confidence interval with 38 subjects such that *G* values within the interval [0.025, 0.027] indicate that all subjects behave similarly. In other words, a subject with a *G* value of more than 0.027 could be considered atypical. Fig. 6B illustrates the mean *G* values over all realizations for each subject, for different group sizes. For each number of subjects *N*_sub_, a threshold can be computed for *G* (see gray curve in Fig. 6B). In addition, the sensitivity of coefficient *G* to the presence of an outlier subject is shown in Figs. 6C and D. For instance, when 1% (Fig. 6C) or 0.2% (Fig. 6D) of voxels of a given subject are atypical, the *G* coefficient identifies it (*G* value of the outlier subject is higher than 0.27). Critically, Fig. 6D suggests that our method can identify outlier subjects even if the outlier effect is present only in a limited number of voxels (e.g., here in 0.2% of 100,000 voxels).

### Object naming data

#### Contribution (global) — *G*

**Fig. 7:**
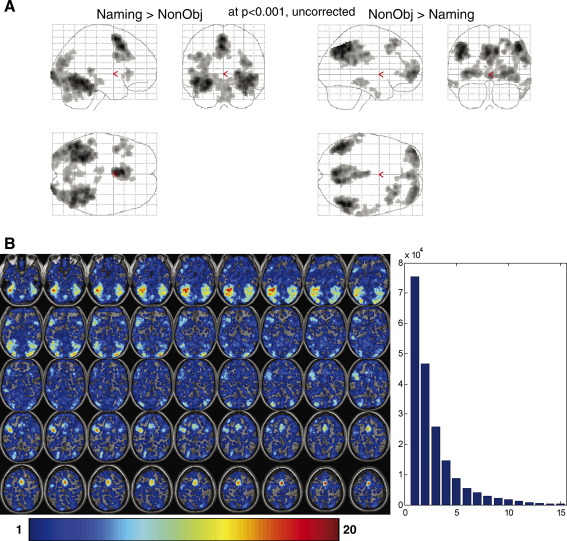
A—(left) group activation for the main effect of object naming relative to the non-object baseline; (right) group activation for the effect of non-object relative to object naming. B—(left) percent of overlap maps that measure how many subjects are activating each voxel at *p* < 0.01 (uncorrected). Voxels observed in one to up twenty subjects are projected as a color-coded map. (Right) Histogram of activated voxels over a given number of subjects. Voxels observed in few subjects (e.g., one or two subjects) represent the majority.

Group activation for the main effect of object naming, relative to the non-object baseline, is shown in Fig. 7A. Positive activations were observed in bilateral fusiform, inferior occipital gyri, cerebellum and SMA, with left lateralized effects in pre-central, inferior frontal, and middle temporal gyri. Negative activations were observed in bilateral inferior and superior parietal regions, precuneus, posterior cingulate and superior frontal gyri, with right lateralized effects in inferior temporal and pre-central gyri. These regions have been observed in previous studies with object naming tasks; see Price et al., 2005 for review. We also illustrate the percentage overlap between thresholded individual maps (Fig. 7B). Basically, these maps represent how often each voxel has been observed as “activated” across subjects at a given individual threshold (*p* < 0.01, uncorrected). The common voxels between subjects are not surprisingly less frequent than voxels that have been observed in only one or two subjects, suggesting that, across subjects, activated regions are variable in size, localization and statistical significance.

**Fig. 8:**
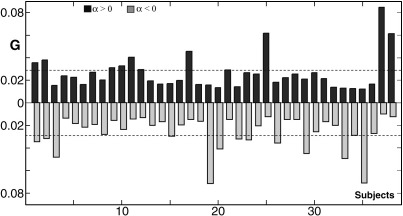
*G* values of all subjects are shown for positive (black bars) and negative (gray bars) *α* value.

Interestingly, the contribution of each subject, as represented by *G*, is variable across subjects (Fig. 8). As demonstrated above using simulated data, given that there were 38 subjects, each subject would be expected to contribute equivalently with *G* values less than 0.027. Some subjects have high *G* values when *α* is positive (e.g., subjects 17, 25, 37, and 38), suggesting that regional activation in these subjects is higher than in the other subjects. For example, subject 37 has a *G* value of 0.09, which suggests that this subject has proportionally higher activation compared to other subjects. Likewise, some subjects have high *G* values when *α* is negative (e.g., subjects 3, 19, 33, and 35), suggesting that these subjects have very low activation in some of the regions activated by the group as a whole. For example, subject 19 has a *G* value of 0.07, which suggests that this subject has a globally low activation level in a relatively large number of voxels, compared to the other subjects.

#### Contribution (local) — *U*

**Fig. 9:**
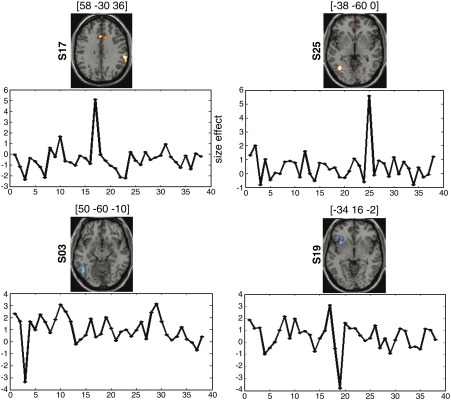
This figure illustrates some of the cortical regions with very different levels of activation in one subject compared to the others. Each functional map (axial view) represents the projection of *U* values (at threshold = 0.3) on a normalized anatomical volume. MNI coordinates of these regions are reported at the top of each functional map. Atypical high activation levels are illustrated for subjects S17 and S25, and atypical low activation levels are illustrated for subjects S03 and S19.

In addition to this global measure, our approach identifies local outliers (at the voxel level). Fig. 9 illustrates some of the cortical regions with very different levels of activation in one subject compared to the others. For instance, subject 17 had higher activation in the right supramarginal gyrus (MNI coordinates: *x* = 58, *y* = − 30, *z* = 36) than all the other subjects. In contrast, subject 3 had lower activation in the left inferior occipito-temporal cortex (*x* = − 50, *y* = − 60, *z* = − 10). These regions have high *U* values (i.e., *U* > 0.9), which suggests that activation in these subjects is very far from the mean of the group.

## Discussion

We have described a new clustering approach, FCP, to identify activations that are driven by one subject relative to the others. Our exploratory analysis for multi-subject fMRI data provides an objective characterization of inter-individual variability. This is usually difficult to achieve by visual inspection alone, particularly when the number of subjects is large. Previous reports have described different ways to discount (i.e., down-weight) the influence of outliers, during first or second-level analyses of fMRI data using, for instance, robust regression approaches (e.g., Diedrichsen and Shadmehr, 2005; Wager et al., 2005). Our method is motivated by the unusual view that outliers are interesting; FCP characterizes variability in individual functional maps to determine whether the variance is meaningful or not. We have illustrated the performance of FCP in a relatively large number of subjects performing an overt naming task. Below, we discuss some potential applications for the analysis of single-case patient studies and the characterization of normal inter-subject variability. We also describe ways that the analysis can be adapted to address specific questions.

### Single-case patient studies

In clinical fMRI, the identification of activations that are driven by patients more or less than groups of control subjects is important, for example, when determining the effect of brain damage on neuronal responses and the mechanisms supporting recovery (e.g., Crinion and Price, 2005; Fernandez et al., 2004; Naeser et al., 2004; Seghier et al., 2001). As there is considerable variability in the site and extent of brain damage, conclusions are often sought on comparing activation in a patient to that in a group of control subjects (e.g., Fernandez et al., 2004; Seghier et al., 2001; Zacks et al., 2004). The sensitivity of conventional parametric statistical tests (e.g., the two sample *t*-test) in these analyses depends on the variance within the control group as well as the degree to which the patient's activation differs from the mean response. If variance in the control group is high (i.e., inflated by outlier values), then atypical activations in the patient may not be detected, even when the patient response lies outside the range of typical responses. We will demonstrate this in an application paper.

Our FCP approach allows a constrained and directed exploration of the data, which can be used to identify where the patient activation pattern is fundamentally different from the regions activated by control subjects. Critically, in this context, FCP facilitates a description of the data in the absence of statistically significant effects, as illustrated here with artificial data (Fig. 6). For example, when a patient has damage to a region that is activated by groups of control subjects, the expectation is that activation in this region will be significantly less in the patient than controls. However, even if the patient activation is atypically low, it will not be significantly different from controls, if the variance within the control group is high. The advantage of using FCP to explore individual variability is that it searches all the voxels (in the SPM{*F*} or the whole brain), rather than being constrained to *a priori* regions of interest. Thus, FCP can identify the full set of regions (neural system) where patient activation is atypically high (or low) compared to the controls. Finally, because identification of regions showing outlier response is based on fuzzy clustering there is no multiple comparison problem (i.e., *U* and *G* are “relative” measures; e.g., Kandel et al., 1995) because there is no categorical declaration of significance of the sort applied to SPMs.

### Atypical activations in healthy populations

There are many sources of variability in normal activation patterns that may not be predicted *a priori* (e.g., Nadeau et al., 1998). These include the availability of different sensorimotor or cognitive strategies for the same task (see Edwards et al., 2005; Noppeney et al., 2004; Price and Friston, 2002; Speer et al., 2003; Tsukiura et al., 2005) and the influence of different behavioral and demographic variables (e.g., Gron et al., 2000; Iaria et al., 2003; Rimol et al., 2006; Rypma et al., 2005). The FCP approach is designed specifically to identify effects that are driven by one or few subjects. It is also useful for highlighting subtle technical problems that were not apparent during pre-processing or first-level statistical analyses. However, FCP is not designed to look at subgroups of subjects that differ in their cognitive approach to a task. Other approaches are being developed for the classification of subgroups including those based on Gaussian Mixture Modeling and Bayesian model comparison procedures (Noppeney et al., 2006; see also Bogorodzki et al., 2005; Zhong et al., 2004). In summary, although our approach is not optimized for classifying subjects into subgroups; it can be used to identify (i) subjects with high *G* values who ‘globally’ drive a significant number of ‘distributed’ voxels, and (ii) subjects with high *U* values who ‘locally’ drive specific cortical regions.

### Adapting the analysis to the question of interest

Our FCP approach is intrinsically parameterized by different factors with fixed numbers of clusters (set to the number of subjects). Principally, the factor *α* is a tuning parameter that allows the user to adjust the sensitivity of the method to outliers (as detailed in Fig. 3). In addition, one can use alternative similarity measures (*D*), including the Pearson correlation distance, the hyperbolic correlation distance (Fadili et al., 2000; Golay et al., 1998) or the Cook distance (Cook and Weisberg, 1982). The choice of the similarity measure is obviously related to the definition of the prototypes, based here on the known high sensitivity and influence of regression analysis to outlier values (Devlin et al., 1975; Stevens, 1984). Likewise, the definition of the degree of contribution of each subject (*G*) can also be reformulated with other measures. The principal motivation here for *G* was to represent, in one measure, how much a subject contributes to the activation at all voxels. Moreover, the quantification of the degree of membership (*U*) could also be modified to include spatial constraints, in particular, to take into account the spatial dependency between each voxel and its neighbors (e.g., Ahmed et al., 2002; Liew et al., 2000).

### Comparison with previous methods

Several approaches have been proposed previously to deal with the presence of outliers. Wager et al. have used robust statistics to down-weight the influence of outliers and assess the mean effects across subjects more accurately (Wager et al., 2005). In the same way, a diagnosis suite, called SPMd, identifies outlier scans at the first (within-subject) level by examining the stability of the fMRI signal over time (Luo and Nichols, 2003) and then removing outlier scans before statistical analysis. This method has been used recently in the context of multi-subject analysis to identify outlier sessions and subjects (Zhang et al., 2006), specifically by computing an “outlier rate” at the global level and exploring the normality of data with the Shapiro–Wilk statistics. With SPMd, it is also possible to generate voxel-specific measures that indicate how far a given subject is from the group mean. The main perspective of these methods is to down-weight or remove effects that are far from other “normal” effects (Luo and Nichols, 2003; Wager et al., 2005). This contrasts with our approach, which “targets” outlier effects in order to characterize them in further analyses. Other approaches have used alternative methods to identify outlier subjects. For instance, Kherif et al. (2003) proposed temporal and spatial similarity measures in order to assess the similarity between subjects before group analysis. This approach, based on a multivariate analysis framework and applied in a multi-contrasts context, assesses the relative inter-subject distance using multidimensional scaling tools. It also uses Cook's test, at the global level, to identify outlier subjects. Moreover, other similarity measures based on independent component analysis (ICA) have been proposed in the context of multi-subject fMRI analysis, including the mutual correlation coefficient between estimated independent components (Esposito et al., 2005) and components from tensor probabilistic independent component analysis (Beckmann and Smith, 2005).

**Fig. 10:**
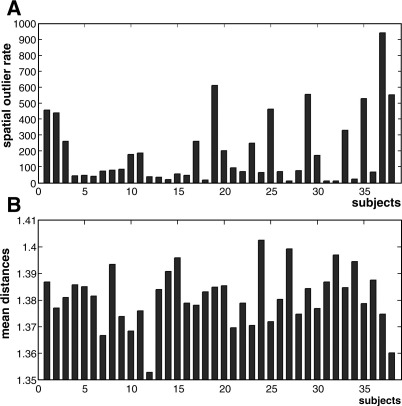
(A) Outlier identification with “SPMd” toolbox of Luo and Nichols (2003). (B) Outlier identification with “distance” toolbox of Kherif et al. (2003).

To compare our FCP approach to others, we re-analyzed our data using SPMd (Luo and Nichols, 2003) and the “distance” toolbox provided by Kherif et al. (2003). With respect to Luo and Nichol's method, we used the recent version “spmd2”, which was developed initially for first (within-subject)-level data diagnosis to ensure the stability of fMRI signals over time. We computed different rates following the multi-subject study of Zhang et al. (2006) and compared the “outlier rate” from SPMd with our global *G* values, as illustrated in Fig. 10A. This demonstrated a number of consistencies between the two approaches. For example, subjects 37, 2, 19, 1, 38 and 29 dominate the activation across voxels, as indicated by our method (Fig. 8). However, we noticed that (i) at the global level, SPMd did not distinguish between outlier effects that are below the mean from those that are above the mean; this may be important when comparing patients to controls, and (ii) there is no quantitative interpretation of this rate, unlike the FCP approach (*G* values under null hypothesis are equal to 1/*N*_sub_, see Fig. 6). SPMd generates a normality diagnostic image based on Shapiro–Wilk statistics. This allows voxels violating normality (outliers) to be identified. Then, individual images are generated to assess how far a subject is from the group mean at a given voxel (equivalent to our *U* value). The assessment of these images by SPMd is based on the assumption that the population effect is normal. In contrast, our FCP does not assume normality.

We also tested the new “distance” toolbox of Kherif et al. (2003) on our 38 subjects. We first displayed the mean distance that indicated how far a given subject is from the group (i.e., the mean of distances between subjects). As shown in Fig. 10B, the mean distance plot suggested that there were no outlier subjects (according also to the Cook test with the default cut off of 0.5). Fig. 10B indicated that some subjects with high mean distance also had high *G* values with our FCP method (e.g., subjects 1, 29 and 33). However, other subjects did not concord with our results (e.g., 8, 24 and 32). The discrepancy is due mainly to the fact that our method is based on a voxel-by-voxel analysis, whereas the approach in the Kherif et al. toolbox is multivariate. Note also that the distance measure employed does not distinguish between effects that are below or above the group mean. Moreover, measures at the voxel level (as in our local measure *U*) are currently not available in this toolbox.

In summary, although outliers can be identified by existing approaches, our approach based on fuzzy set theory is fundamentally different because (i) it acts directly on second (between-subject)-level summary statistics; (ii) it uses fuzzy logic theory which may be more appropriate for vague and ambiguous concepts like typicality and outliers; (iii) it models all subjects explicitly as potential outliers by fixing the prototypes, (iv) it employs a robust local similarity measure (*D*) at the voxel level that can be adapted easily to other contexts; (v) it provides a way to identify outlier subjects (i.e., with the *G* value) at the global level and specifies if this outlier effect is below or above the group mean (i.e., the sign of parameter *α*); and (vi) it furnishes a local measure (i.e., a *U* value) that allows voxels with atypical activation levels to be identified in each subject.

## Conclusion

Here, we have presented a new approach that identifies subject-driven activations in fMRI data. This method could be used as an exploratory approach in multi-subject fMRI studies. Its sensitivity is illustrated here with both synthetic and real data from a relatively large number of healthy right-handed subjects performing an object naming task. Future investigations will explore the specificity of such approaches in group studies when activation is expected to be heterogeneous, for example in left-handed, multilingual, pediatric or diseased populations.

## Footnotes

1 In statistics, observations that are far outside the norm of a population have been described under different names, including unrepresentative, influential, “rogue”, contaminant, “maverick”, outlier or fringe-lier observations. For the rest of the paper, we will use the term “outlier” to designate this kind of observation.
